# Self-assessed mental health problems and work capacity as determinants of return to work: a prospective general population-based study of individuals with all-cause sickness absence

**DOI:** 10.1186/1471-244X-13-259

**Published:** 2013-10-14

**Authors:** Gunnel Hensing, Monica Bertilsson, Gunnar Ahlborg, Margda Waern, Marjan Vaez

**Affiliations:** 1 Department of Social Medicine, The Sahlgrenska Academy at the University of Gothenburg, Göteborg, Sweden; 2 Department of Occupational and Environmental Medicine, The Sahlgrenska Academy at the University of Gothenburg, Göteborg, Sweden; 3 Department of and Psychiatry and Neurochemistry, The Sahlgrenska Academy at the University of Gothenburg, Göteborg, Sweden; 4 Divisionof Insurance Medicine, Department of Clinical Neuroscience, Karolinska Institutet, Stockholm, Sweden; 5 Institute of Stress Medicine, Region Västra Götaland, Gothenburg, Sweden

**Keywords:** Self-reported mental health problems, Self-assessed work capacity, Return to work, Sickness absence

## Abstract

**Background:**

Mental health problems are common in the work force and influence work capacity and sickness absence. The aim was to examine self-assessed mental health problems and work capacity as determinants of time until return to work (RTW).

**Methods:**

Employed women and men (n=6140), aged 19–64 years, registered as sick with all-cause sickness absence between February 18 and April 15, 2008 received a self-administered questionnaire covering health and work situation (response rate 54%). Demographic data was collected from official registers. This follow-up study included 2502 individuals. Of these, 1082 were currently off sick when answering the questionnaire. Register data on total number of benefit compensated sick-leave days in the end of 2008 were used to determine the time until RTW. Self-reported persistent mental illness, the WHO (Ten) Mental Well-Being Index and self-assessed work capacity in relation to knowledge, mental, collaborative and physical demands at work were used as determinants. Multinomial and binary logistic regression analyses were used to estimate odds ratios with 95% confidence intervals (CI) for the likelihood of RTW.

**Results:**

The likelihood of RTW (≥105 days) was higher among those with persistent mental illness OR= 2.97 (95% CI, 2.10-4.20) and those with low mental well-being OR= 2.89 (95% CI, 2.31-3.62) after adjusting for gender, age, SES, hours worked and sick leave 2007. An analysis of employees who were off sick when they answered the questionnaire, the likelihood of RTW (≥105 days) was higher among those who reported low capacity to work in relation to knowledge, mental, collaborative and physical demands at work. In a multivariable analysis, the likelihood of RTW (≥105 days) among those with low mental well-being remained significant OR=1.93 (95% CI 1.46-2.55) even after adjustment for all dimensions of capacity to work.

**Conclusion:**

Self-assessed persistent mental illness, low mental well-being and low work capacity increased the likelihood of prolonged RTW. This study is unique because it is based on new sick-leave spells and is the first to show that low mental well-being was a strong determinant of RTW even after adjustment for work capacity. Our findings support the importance of identifying individuals with low mental well-being as a way to promote RTW.

## Background

Among the factors that can negatively affect capacity to work, mental health problems play a significant part. They are common in the general population and even minor illnesses can influence the capacity to work to a great extent [1-4]. Common mental disorders (CMD) is an encompassing term for mild to moderate depression, anxiety disorders and mental exhaustion/burnout including subthreshold symptoms. CMD is often associated with significant dysfunction and disability and co-morbidity is common. CMD have been found to have a stronger impact on sickness absence and work impairment than breathing disorders, back/neck disorders, arthritis, diabetes and heart disease [2,4,5].

Several studies have examined the role of CMD in the process of return to work (RTW). Brouwers et al. [6] found that a long duration of mental health problems before the start of sick leave caused by minor mental disorders and a long duration of sick leave before inclusion in the study reduced the probability of RTW within 3 months. High levels of somatization and anxiety at baseline and depression after 3 months reduced the probability of RTW even further [6]. In another Dutch study, Brouwer et al. [7] found that the only factor that remained significantly associated with time until RTW among those with mental health problems was willingness to expend effort in completing behaviour (adjusted for gender, age, level of education, time to identification by the occupational health service, intensity of medical condition causing the sick leave, and perceived social support). In a prospective study involving a multivariable analysis of time to RTW, Vlasveld et al. [8] found that 71% of the workers initially off sick had returned to full RTW within 1 year. High physical job demands, contact with medical specialists, high level of physical symptoms, moderate to severe depressive symptoms and older age were associated with prolonged sick leave. In 2012, Roelen et al. [9] found that the association between mental health problems and RTW varied between different mental disorders. In a systematic and comprehensive review, Cornelius et al. [10] found strong evidence that age >50 years prolonged the time off work. Limited evidence was found for any associations between other individual factors, such as gender, education, history of previous sickness absence, negative recovery expectation and socioeconomic status, and disability and time off work. Health-related factors (stress, shoulder/back pain and depression/anxiety disorder) and job-related factors (unemployment, quality and continuity of occupational care and supervisor behaviour) were also associated with increased disability and slow RTW. Cornelius et al. [10] concluded that there is a need for further research, particularly prospective cohort studies.

An important aspect of RTW is work capacity. Work capacity, or work ability which also is a common term, can be understood as the individual’s capacity to do his/her work with respect to the demands at work, given a certain health status and mental resources [11]. Thus, there is an interaction between the individual and his/her work environment. It can be seen as a dynamic relationship between the individual and different aspects of the work environment including work tasks [12]. However, none of the studies mentioned earlier included measures of work capacity. From a clinical perspective and to identify individuals in need of specific support or treatment at an early stage, it is important to know the determinants of time until RTW. Thus, the aim of this general population-based, prospective cohort study was to examine self-assessed mental health problems and work capacity as determinants of time until RTW.

## Methods

### Study design and participants

This prospective cohort study is part of the Health Assets Project [13,14]. The study base was the region of Västra Götaland in southwest Sweden with a population of 1.6 million (17% of the Swedish population). The region includes both urban and rural areas. The target population included individuals aged from 19 to 64 years.

Individuals who became sick-listed with a new period of sick leave at the Swedish Social Insurance Agency between February 18 and April 15, 2008, irrespective of the reasons for the sick leave (all-cause sickness absence) were identified as a relevant study population. The time period were chosen to avoid seasonal variations where sickness absence in Sweden is increased due to influenza periods or decreased due to summer vacation. The chosen months represented the annual mean. During the time period of inclusion a total number of 12, 543 individuals became sick-listed. Of these 49% (*n*=6140) were reported as off sick by their employer and registered as such at the Swedish Social Insurance Agency between the February 18 and April 15, 2008. This group was invited to the study and they all received a questionnaire. The remaining 51% (*n*=6403) became sick-listed during the same period but were either not reported by their employer as off sick or not registered as off sick by the Swedish Social Insurance Agency (or both) until after April 15, 2008. Those who were registered at a later date were not invited to participate in this questionnaire study, because we aimed to distribute the questionnaire as close as possible to the actual sick-leave period. Among those registered after April 15 and thus not included in this study, there was a higher proportion of men, individuals with low income, highly educated, first time sick-listed and immigrants, according to oral information from the Department for Statistics and Analysis at the Swedish Social Insurance Agency.

Postal questionnaires to the group (n=6140) registered between February 18 and April 15, 2008 were distributed by Statistics Sweden at base line (2008) and two reminders followed [15]. The response rate was 54% (n=3310). Significantly higher dropout rates were found among persons who were young (aged 19–30 years), single, born outside Sweden and those reporting low annual income (≤SEK149,000/year which corresponds to approximately 17,380 EURO). In urban areas, women were more likely to drop out than men. The proportion of women (66%) and men (34%) among those who responded to the questionnaire was similar to that observed for sickness absence in the general population of the country as a whole [16].

Of those who decided to participate by answering the questionnaire (54%, *n*=3310) we included in this follow-up study individuals with only a single sick-leave spell by the end of 2008 and who stated in the questionnaire that they were employed (*n1*=2502). Register data on sickness absence was followed until the end of 2008. Sickness absence is dynamic in the sense that individuals move in and out of the state of being off sick. In this study, there was, by necessity, a delay between the start of the inclusion period and the date of completion of the postal questionnaire. Thus, in the second part of this study we included only those who stated that they were currently on sick leave at the time of answering the questionnaire (*n2*=1802) to be sure that self-assessment of work capacity was done during a sick leave episode among all participants. This sub sample (*n2*) did not differ from the larger sample (*n1*) or from the original sample (*n*=3310) with regard to socio-demographic factors included. There were a higher proportion of individuals with persistent mental illness and with low mental wellbeing in the sub sample (*n2*) compared to the larger sample (*n1*) but this was expected since individuals with mental health problems have an increased risk for longer periods off work. Thus, in any sample followed over time the proportion with mental illness will increase.

### Social insurance in Sweden

In Sweden, sickness absence insurance covers all inhabitants of working age. During the study period, sickness benefit amounted to approximately 80% of lost income up to a certain level. Sickness benefit can be granted to those who have impaired work capacity due to disease or injury. There is no economic reimbursement for the first qualifying day. The initial 7 days in a sick-leave spell are self-certified. After that, a medical certificate is required. Sick pay is covered by the employer for the first 14 days of a sick-leave spell. Thereafter, sick-leave benefit is granted from the Swedish Social Insurance Agency.

### Outcome: time until RTW

In this study, time until RTW was measured by estimating the number of sick-leave days for which sickness benefit was paid in 2008. Information on an individual’s annual number of sick-leave spells and number of benefit-compensated sick-leave days was collected from the longitudinal integrated database for sickness insurance and labour market research (LISA), held by Statistics Sweden. The total number of benefit-compensated sick-leave days in 2008 was divided into three groups: ≤14 days (*n*=996), 15–90 days (*n*=913) and ≥91 days (*n*=593). All participants had an initial 14-day period compensated by sick pay from the employer, therefore the total number of actual sick-leave days was by adding 14 days per person. Thus, the final definitions of RTW were early (≤28 days), medium late (29–104 days) and late (≥105 days). Early RTW was chosen as a relevant category in line with several other studies, given the fact that all participants had at least 2 weeks off work as part of the inclusion criteria [6,17,18]. Medium late RTW was harmonized with the Swedish sickness insurance regulation whereby there is an obligatory assessment of the individual’s work capacity in relation to available work tasks at the work place at 3 months. Late RTW was defined so that enough individuals were included to allow relevant statistical analyses and was not divided into further categories. In the analysis of the subpopulation currently on sick leave, time until RTW was treated as a binary outcome: early/medium late (≤104 days) versus late RTW (≥105 days). This dichotomization was done due to the smaller number of individuals.

### Indicators of mental health problems

Two self-reported indicators of mental health problems were used. Respondents were asked whether they had any persistent disease, illness or disability, followed by a checklist of disease categories. Those who ticked “mental illness” were considered to have persistent mental illness. This question, which has been used extensively in different public health surveys in Sweden, has shown good validity and reliability [19]. Mental well-being was assessed using the WHO (Ten) Mental Well-Being Index, which has been used in several population-based studies with acceptable validity and reliability [20-22]. In comparisons with clinical interviews using the Schedules for Clinical Assessment in Neuropsychiatry, the WHO (Ten) Mental Well-Being Index was found to be more likely to identify need of care and psychiatric diagnoses than the other instruments [23,24]. The index measures mental well-being during the previous week and includes one item on depression and anxiety, two items on energy and six items on positive well-being. Response alternatives to each item are always, often, sometimes and never. The maximum score is 30 points, and higher scores indicate higher mental well-being. In the current study, we computed the cut-off based on the lower quartile in a general population cohort available in the Health Assets Project. Thus, the cut-off was not computed on the population included in the present study because that consists of sick-listed individuals and the distribution might be skewed. The cut-off was chosen to capture enough exposure differences, without having to compare the extremes. Two status categories were created, namely low mental well-being (scores ≤12) and high mental well-being (scores ≥13). The mental health variables overlapped; among those reporting persistent mental illness (n1) 71% had low mental well-being and of those reporting low mental well-being 22% stated persistent mental illness.

### Self-assessed work capacity

Work capacity was measured by an initial question and four separate items reflecting different aspects of work capacity: “How do you rate your current work ability with respect to knowledge, mental, collaborative and physical demands of your work?” (each item assessed separately). The mental and physical demands items were derived from the Work Ability Index (WAI) [25]. Psychometric evaluation of the WAI has shown that these two items correlate highly with the total index [26]. The knowledge demands item was derived from the Copenhagen Psychosocial Questionnaire [27]. The collaborative demands item was developed by the research group [28,29]. Response alternatives for each item were very good, rather good, moderate, rather poor and very poor. Response alternatives were dichotomized into high work capacity (very good and rather good) and low work capacity (moderate, rather poor, very poor). Dichotomization was done mainly to attain analytical power in subgroups. The correlation between physical work capacity and the other work capacity variables was in each comparison below r=0.40. The correlation between mental work capacity and work capacity related to knowledge demands was r=0.53. The correlations between collaborative work capacity and mental and knowledge work capacity were r=0.60 and r=0.52. All correlations were significant at the 0.01 level (Spearman’s correlation).

### Confounders

Data on gender, age, socioeconomic status (SES), and total number of sick-leave days during the calendar year 2007 were obtained from national registers. Age was categorized into three groups: 19–30, 31–50 and 51–64 years. SES was defined according to a classification system used by Statistics Sweden, which is based on occupation [30]. Each person was assigned to one of three groups: high non manual, intermediate/low non manual and skilled/unskilled manual/self-employed. Previous sick leave was defined as having at least one sick-leave day with benefit from the National Insurance Agency during the year before inclusion (2007). This implies a period of at least 15 days of sick leave according to the Swedish insurance system, because shorter periods are registered by the employers only. Data on marital status, educational level and hours worked were obtained from the questionnaire. Marital status was grouped into married/cohabiting or single. Educational level was categorized as up to primary (9 years or less), upper secondary (10–12 years) and higher education (>12 years). Hours worked were categorized into full-time and part-time (at least 15 hours/week).

Because there is a high comorbidity between mental health problems and alcohol problems, a separate analysis was made [31]. The associations between mental health problems and alcohol problems were analysed by using Pearson's chi^2^ with the significance level set at p<0.05. As an indicator of harmful alcohol habits, the Swedish version of the WHO’s recommended questionnaire the AUDIT (Alcohol Use Disorders Identification Test) was used [32]. Significant associations were found between AUDIT scores, persistent mental illness and low mental well-being. However, AUDIT scores were not significantly associated with RTW in any of the stratification groups: age, gender, persistent mental illness and low mental well-being (results not shown). Harmful alcohol habits (AUDIT scores) were thus not included in any further analysis.

The study was approved by the Regional Ethical Review Board of the University of Gothenburg, Sweden (no. 039–08).

### Statistical analyses

For the WHO (Ten) Mental Well-Being Index, 198 individuals (7.9% of the study population) had missing values for at least one item. Those who had missing values on all items (*n*=5) were excluded; missing data for the remaining individuals (*n*=193) were replaced by mode imputation at each item to increase power [33]. After imputation, the proportion reporting high or low mental well-being did not change in the study population or in the subpopulation of those currently off sick.

Multinomial logistic regression was used (*n*=2502) to estimate crude and adjusted odds ratios (OR) with 95% confidence intervals (CI) for likelihood of time until RTW (as categorical outcome) with respect to independent variables. Despite non significance in the bivariate analyses, gender and SES were included in the multivariable analyses because of their known strong association with both mental health problems and sickness absence.

Binary logistic regressions were performed (*n*=1082) to estimate crude and adjusted (age, gender, persistent mental illness, low mental well-being) ORs with 95% CIs for late RTW (as a binary outcome) in relation to knowledge, mental, physical and collaborative work capacity among the subpopulation of those currently off sick. Other independent covariates were not included due to non significant association with late RTW or work capacity variables (results not shown). The group reporting high work capacity was used as reference category.

The analyses were made using IBM SPSS Statistics 20.

## Results

### Determinants of RTW

The characteristics of the study population and associations with RTW are presented in Table 1. No significant differences in RTW were found between men and women. Among men, 40% had an early, 38% had a medium late (29–104 days) and 22% a late (≥105 days) RTW (Table 1); the corresponding figures for women were 40%, 36% and 24%. The two older age groups had increased odds for late RTW compared with those aged 19–30 years but no differences were found for medium late RTW. A larger proportion of those working part-time had a late RTW compared with those working full-time (OR 1.39, 95% CI 1.11–1.74).

**Table 1:** **Bivariate multinomial logistic regression analyses with crude odds ratio (OR) with 95**%** confidence interval (CI) for medium and late RTW compared with early RTW** (***n***=**2502**, **856 men and 1646 women**), **Health Assets Project 2008**

**Independent variables**	**RTW**					
	**Medium late RTW (29**–**104 days)**			**Late RTW (≥105 days)**		
	***n *** **(exp)**	**% (exp)**	**OR (95% CI)**	***n *** **(exp)**	**% (exp)**	**OR (95% CI)**
Gender						
Men	322	37.6	1	192	22.4	1
Women	591	35.9	0.96 (0.80–1.16)	401	24.4	1.09 (0.88–1.36)
Age						
19–30 years	115	52.1	1	41	15.0	1
31–50 years	396	35.5	0.90 (0.67–1.20)	271	24.3	1.72 (1.70–2.54)
51–64 years	402	36.1	0.95 (0.71–1.27)	281	25.2	1.87 (1.27–2.74)
Marital status						
Married/cohabiting	675	37.1	1	430	23.6	1
Single	22	34.3	0.88 (0.72–1.08)	158	24.4	0.98 (0.78–1.24)
Educational level						
Higher education	303	35.9	1	200	23.7	1
Upper secondary education	383	36.0	1.01 (0.89–1.24)	252	23.7	1.00 (0.80–1.27)
Primary education	215	38.2	1.12 (0.88–1.43)	132	23.4	1.04 (0.79–1.38)
Socioeconomic status						
High nonmanual	113	38.6	1	79	27.0	1
Intermediate/low nonmanual	297	34.2	0.73 (0.54–0.99)	208	23.9	0.73 (0.52–1.03)
Skilled/unskilled workers and self-employed	484	37.0	0.83 (0.62–1.12)	303	23.2	0.74 (0.54–1.03)
Hours worked						
Full-time	673	37.6	1	388	21.7	1
Part-time	220	33.6	0.95 (0.77–1.76)	185	28.2	1.39(1.11–1.74)
Past year sick-leave days						
No	704	36.0	1	436	22.3	1
Yes	209	38.1	1.32 (1.06–1.65)	157	28.6	1.60 (1.26–2.04)
Persistent mental illness						
No	840	37.1	1	494	21.8	1
Yes	73	31.1	1.29 (0.91–1.83)	99	42.1	2.97 (2.13–4.15)
Mental well-being						
High	698	39.6	1	308	17.5	1
Low	214	29.2	0.98 (0.80–1.22)	284	38.7	2.96 (2.38–3.68)

Sickness absence days in 2007 were associated with medium late RTW (OR=1.32, 95% CI 1.06–1.65) and late RTW (OR=1.60, 95% CI 1.26–2.04). Self-reported persistent mental illness (OR= 2.97, 95% CI 2.13–4.15) and low mental well-being (OR 2.96, 95% CI 2.38–3.68) were significantly associated with late RTW but not with medium late RTW. No significant associations with medium late or late RTW were found for marital status, educational level and SES.

The association between persistent mental illness and late RTW remained significant after adjustment for other covariates (Table 2). The same was the case for the association with low mental well-being. The strongest association was found between persistent mental illness and late RTW (OR=2.97, 95% CI 2.10–4.20).

**Table 2:** **Adjusted odds ratio (OR) with 95**%** confidence interval (CI) for medium and late RTW compared with early RTW in relation to mental health problems for all participants in the cohort** (***n***=**2502**), **based on multiple multinomial logistic regression analyses**, **Health Assets Project**, **2008**

	**Time until RTW**			
	**Medium late RTW (29–104 days)**		**Late RTW (≥105 days)**	
	**Model 1: OR**	**Model 2: OR**	**Model 1: OR**	**Model 2: OR**
	**(95% CI)**^**a**^	**(95% CI)**^**b**^	**(95% CI)**^**a**^	**(95% CI)**^**b**^
**Persistent mental illness**				
Yes (reference: no)	1.33 (0.93–1.91)	1.29 (0.90–1.86)	3.08 (2.18–4.35)	2.97 (2.10–4.20)
**Mental well**-**being**				
Low (reference: high)	0.98 (0.78–1.21)	0.96 (0.77–1.20)	2.94 (2.34–3.67)	2.89 (2.31–3.62)

^a^Adjusted for gender, age, socioeconomic group and hours worked.

^b^Adjusted for model 1 and sick-leave days in 2007.

### Work capacity as a determinant of RTW among employees currently off sick

Among those who currently were off sick (see methods section) when responding to the questionnaire, 54% of the men and 55% of women had a late RTW. In each of the four work capacity dimensions, those who rated their capacity to work as low had higher odds of late RTW compared with those with high work capacity (Table 3).

**Table 3:** **Crude and adjusted odds ratio (OR) with 95**%** confidence interval (CI) for late RTW compared with early**/**medium late RTW among individuals currently on sick leave** (***n***=**1082**, **354 men and 728 women**) **with regard to work capacity**, **adjusted for persisting mental illness and mental well**-**being**: **results of binary logistic regression analyses**, **Health Assets Project 2008**

**Independent variable: work capacity in relation to demands at work**	***n *** **(exposed)**	**Late RTW (≥105 days)**						
		**%**	**Crude OR (95% CI)**	**Model 1: OR(95% CI)**^**a**^	**Model 2: OR (95% CI)**^**b**^	**Model 3: OR (95% CI)**^**c**^	**Model 4**: **OR (95% CI)**^**d**^	**Model 5: OR (95% CI)**^**e**^
*Knowledge demands*								
High	451	68.3	1	1	1	1	1	1
Low	112	51.3	2.04 (1.43–2.91)	2.06 (1.44–2.95)	1.91 (1.33–2.75)	1.31 (0.85–2.02)	1.75 (1.21–2.53)	1.34 (0.87–2.06)
*Mental demands*								
High	335	48.8	1	1	1	1	1	1
Low	225	64.1	1.87 (1.44–2.44)	1.93 (1.47–2.53)	1.79 (1.35–2.39)	1.35 (0.96–1.89)	1.52 (1.14–2.04)	1.17 (0.83–1.65)
*Collaborative demands*								
High	408	50.3	1	1	1	1	1	1
Low	153	67.4	2.04 (1.50–2.78)	2.04 (1.49–2.79)	1.90 (1.37–2.62)	1.34 (0.90–2.00)	1.71 (1.23–2.36)	1.30 (0.87–1.94)
*Physical demands*								
High	295	49.9	1	1	1	1	1	1
Low	273	60.1	1.51 (1.18–1.94)	1.53 (1.19–1.97)	1.50 (1.17–1.93)	1.25 (0.95–1.64)	1.44 (1.12–1.86)	1.24 (0.94–1.28)

^a^Adjusted for gender and age.

^b^Adjusted for gender, age and persisting mental illness.

^c^Adjusted for gender, age, persisting mental illness, and the other three work capacity items.

^d^Adjusted for gender, age and mental well-being.

^e^Adjusted for gender, age, mental well-being, and the other three work capacity items.

Among individuals with low work capacity, the age- and gender-adjusted OR of late RTW was 2.06 (95% CI 1.44–2.95) in relation to knowledge demands, 1.93 (95% CI 1.47–2.53) for mental demands, 2.04 (95% CI 1.49–2.79) for collaborative demands and 1.53 (95% CI 1.19–1.97) for physical demands. After adjustment for persistent mental illness, ORs were reduced for each dimension but remained significant (Table 3).

Although ORs were further reduced in the model adjusting for low mental well-being, associations remained significant. However, when all the different work capacity dimensions were included in the final model, the associations became non significant.

### Mental health problems as determinants of RTW among employees currently off sick

The proportion with late RTW was 38% (*n*=41) among those aged 19–30 years, 56% (*n*=267) among those aged 31–50 years and 56% among those aged 51–64 years (*n*=280). The likelihood for late RTW among the two older age groups compared with the youngest was OR 2.04 (95% CI 1.33–3.13) and OR 2.08 (95% CI 1.35–3.19), respectively.

Odds for late RTW were higher among those with persistent mental illness (OR 1.75, 95% CI 1.21–2.52) also after adjusting for age and gender. An adjustment was made for each of the four dimensions of work capacity and odds were reduced (Table 4). The adjustment for mental work capacity led to a non significant OR and for collaborative work capacity, the OR was just above the significance level. When all four dimensions of capacity to work were included in the model, the association between persistent mental illness and late RTW became non significant (Table 4). Among those with low mental well-being, the OR for late RTW was 2.18 (95% CI 1.69–2.82) after adjusting for age and gender. The same procedure was used here and adjustment for each dimension of work capacity was made separately. This yielded only minor changes in the ORs. A significant two-fold increase in odds was noted in all cases. After adjustment for all capacity to work dimensions the likelihood for late RTW among employees with low mental well-being was still increased (OR 1.93, 95% CI 1.46–2.55).

**Table 4:** **Crude and adjusted odds ratio (OR) with 95**%** confidence interval (CI) for late RTW compared with early**/**medium late RTW among individuals currently on sick leave** (***n***=**1082**, **354 men and 728 women**) **with regard to mental health problems adjusted for work capacity**: **results of binary logistic regression analyses**, **Health Assets Project 2008**

**Independent variable**	***n *** **(exposed)**	**Late RTW (≥105 days)**							
		**%**	**Crude OR (95% CI)**	**Model 1: OR (95% CI)**^**a**^	**Model 2: OR (95% CI)**^**b**^	**Model 3: OR (95% CI)**^**c**^	**Model 4: OR (95% CI)**^**d**^	**Model 5: OR (95% CI)**^**e**^	**Model 6: OR (95% CI)**^**f**^
*Persistent mental illness*									
No	489	52.6	1	1	1	1	1	1	1
Yes	99	65.1	1.69 (1.18–2.41)	1.75 (1.21–2.52)	1.60 (1.09–2.35)	1.38 (0.93–2.06)	1.50 (1.01–2.20)	1.71 (1.17–2.49)	1.37 (0.92–2.05)
*Mental well*-*being*									
High	303	46.8	1	1	1	1	1	1	1
Low	284	65.6	2.16 (1.68–2.78)	2.18 (1.69–2.82)	2.10 (1.64–2.74)	1.97 (1.50–2.60)	2.0 (1.53–2.62)	2.14 (1.64–2.78)	1.93 (1.46–2.55)

^a^Adjusted for age and gender.

^b^Adjusted for age, gender and work capacity in relation to knowledge demands.

^c^Adjusted for age, gender and work capacity in relation to mental demands.

^d^Adjusted for age, gender and work capacity in relation to collaborative demands.

^e^Adjusted for age, gender and work capacity in relation to physical demands.

^f^Adjusted for age, gender and work capacity in relation to knowledge, mental, collaborative and physical demands.

## Discussion

To the best of our knowledge, this is the first study to examine both mental health problems and work capacity in relation to RTW in a general population-based cohort of employees off sick due to all-cause sickness absence. Low mental well-being was a strong determinant with a near three-fold increase in odds of late RTW in the adjusted model. Similar odds were found in a separate model assessing the relationship between self-reported persistent mental illness and RTW. Another important finding was that each of the four work capacity dimensions (knowledge, mental, collaborative and physical) predicted late RTW also in models adjusted for age, gender, persistent mental illness and low mental well-being, respectively. Somewhat unexpectedly, low mental well-being remained a significant determinant of late RTW after adjustment for all four work capacity dimensions.

### Determinants of RTW

We found that individuals who reported persistent mental illness had a higher likelihood of late RTW. Our study lacks specific diagnostic information, but it can be assumed that many of those with self-reported persistent mental illness had anxiety and/or depressive disorders. Individuals in this study were initially off sick and the disease that led to the sick-leave episode might have triggered a relapse or worsening of mental illness. Furthermore, individuals with persistent mental illness might be more vulnerable to strain associated with the sick-leave episode, be it health or work related. Conditions at work might influence RTW and several studies have shown that mental illness is associated with stigma [34]. It is possible that individuals with mental illness refrain from disclosing their problems and this might reduce the access to vocational support needed in the RTW process [34]. It is well known from previous studies that individuals with mental health problems to a large extent go unidentified at both primary health care centres and specialized medical clinics [35,36]. From the current study, it is not clear whether persistent mental illness was detected or not. A medical consultation is a prerequisite for sickness benefit after 7 days, and all individuals in this study were thus seen by a doctor. However, the extent to which symptoms of CMD were identified and treated could not be determined in our study. In one of the few studies estimating undetected CMD in a national register of all-cause long-term sickness absence (exceeding 8 weeks), Soegaard [36] found that, among individuals without any psychiatric sick-leave diagnosis, 19.9% had undetected depression and 6.4% had undetected anxiety symptoms [36].

A more unexpected finding was that even after adjustment for all dimensions of capacity to work, low mental well-being was associated with late RTW. A Swedish population-based study concluded that very mild psychological distress at baseline, measured by the General Health Questionnaire (GHQ 12), had a hazard ratio of 1.7 for a disability pension for a somatic reason and a hazard ratio of 2.2 for a disability pension with a psychiatric reason 5 years later [37]. Our study has a shorter follow-up period, but the findings are in the same direction. Thus, it seems important to identify low mental well-being to avoid lingering absence and, in the longer-term perspective, disability pensions. In psychiatric research, it has been suggested that well-being is an important complement to diagnostic procedures of symptoms because it has higher relevance to an individual’s quality of life, and it may better capture recovery or subclinical symptoms [35,38,39].

Our study sample consisted of individuals off sick with all-cause sickness absence and low mental well-being might be secondary to severe acute or chronic disorders such as injuries, circulatory diseases or neoplasms. Psychiatric disorders constituted the most common diagnostic group among sick-listed women in Sweden in 2008, accounting for 33% of all cases of sickness absence. In men, psychiatric disorders were the second most common diagnostic group, and they accounted for 23% of all cases [16]. The diagnostic panorama changes as the sick-leave periods become longer because most individuals with infectious diseases and injuries return to work at a faster pace. Thus, the study population is mixed over all diagnostic groups and this needs to be taken into account when interpreting the findings. It is likely that physicians identify low mental well-being more easily in individuals who primarily seek care for mental symptoms, and less easily in individuals who primarily seek care for other causes. Work capacity regarding knowledge, mental, collaborative and physical demands predicted RTW in our study. There are no previous studies that can provide data for direct comparison.

In a clinical population from primary and occupational health care, Reiso et al. [17] used a single question: “to what degree is your ability to perform your ordinary, remunerative work reduced today?”. They found that individuals who reported that their ability to perform work was very much reduced had significantly prolonged sickness absence at follow-up at 1 and 3 months. The single question “current work ability compared with life-time best” was used in a study investigating RTW in a cohort of newly sick-listed patients in primary and occupational health care [18]. Patients with musculoskeletal disorders and those with mental disorders improved their work ability assessments but significant association with RTW was found only for those with musculoskeletal disorders [18]. This is in line with our findings that low mental well-being was a strong determinant for late RTW. On the other hand, it might be that work capacity assessments are more complex both in the clinical setting and as self-assessments. A recent qualitative study explored the capacity to work while depressed and anxious and identified a dynamic and varying capacity related both to fluctuations in symptoms and to work tasks and work environment. Even the social environment and life outside work were highlighted as important for the capacity to work [40]. New instruments or items need to be developed to better capture the complexity of the phenomenon in a population-based study but probably also in the clinical setting. Niewenhuijsen et al. [41] concluded in a review that there was a lack of functioning instruments detecting the very specific deficits in mental disorders.

### Practice implications

The clinical implications from this study should be regarded from a sickness absence perspective. It might be important to develop easily administered screening questions to identify individuals in need of more intense interventions with the aim of reducing time to RTW. Several future studies are needed but, from this study, it seems more important to screen for mental well-being than for work capacity assessment. The length of sickness absence should be adapted to the individual resources and work demands and, in some cases, a longer period of absence is needed, relevant and well-motivated. However, to be sick-listed might lead to negative and unwanted consequences such as reduced work motivation, social isolation, stigmatization, changed self-image, economic strain and secondary health problems. National sickness insurance regulations must also be considered and the way they might affect individuals on prolonged sick leave. In Sweden, for example, a new and stricter sickness insurance regulation means that after 3 months sick leave, a work place transfer to another job at the same employer but with lesser demands must take place. After 6 months of sick leave, if the person cannot return to work, a new job on the open labour market is the alternative. Regulations like this increase the pressure on the individual and the employer to make work possible and there is a large responsibility also for the health care to be efficient and professional in support of sick-listed individuals. Early identification of mental health problems is an important component in this process.

### Methodological considerations

The strengths of this study are the selection of a general population-based consecutive sample of newly sick-listed individuals obtained from national registers, the use of two different questions to capture mental health problems, and the inclusion of different dimensions of work capacity. The outcome measure, RTW, was prospective and the register data were of high quality.

There are some limitations to discuss. Different types of bias can occur from self-assessed measurements including social desirability, recall bias and responses biased by symptoms associated with mental health problems (e.g. depression). Validated and recommended instruments have been used for the measurement of health-related variables [20,24]. The validity of the WHO (Ten) Mental Well-Being Scale was tested in the PART project; a population-based study from Sweden found that the WHO (Ten) Mental Well-Being Index was more likely to identify individuals in need of care and psychiatric diagnosis compared with diagnostic questionnaires on CMD, which were seen as too inclusive [24]. Mental and physical capacity to work was measured by validated instruments [25,42]. However, knowledge and collaborative work capacity items were developed within the project and have not been tested for validity. Even though the associations were similar to those found for mental and physical work capacity, we cannot claim that these new constructs are equally valid. It can be argued that social desirability played a role in the assessments of collaborative work capacity; the second lowest proportion reporting low work capacity was found for this dimension. It is likely that an overestimation of one’s own capacity to collaborate is more common than an underestimation. Regarding memory and symptom bias, it can be argued that individuals with CMD might undervalue their capacities to a greater extent than the mentally healthy. However, the findings in this study were quite strong and remained significant even after adjustments, so even though there might be a slight overestimation of a specific OR, the direction would be the same with some changes in the level of estimations of, for example, capacity to work.

There was some dropout in the first phase of inclusion in this study. Not all individuals who actually became sick-listed between February 18 and April 15, 2008, were registered as off sick during that period. In our study, 49% were included, which is a large proportion of the target population. Multivariable analyses controlling for possible confounders such as gender, educational level, SES, and total number of sick-leave days during the year before to the index episode were done as a way to manage any bias related to overrepresentation of certain groups among those registered after April 15.

There was also a systematic dropout in the questionnaire study and the estimates need to be interpreted with this in mind. The dropout was higher among younger persons. From a sick-leave perspective, this means that a group with low risk for long-term sick leave had a higher dropout rate. The proportion for early RTW might have been higher if the age-related dropout had not occurred. On the other hand, young individuals with mental health problems might have more problems related to the capacity to work than older individuals due to their lack of experience of work life. However, this would strengthen the overall association between RTW and persistent mental illness and low mental well-being rather than attenuate it. The lower representation of immigrants most likely led to an underestimation of associations because mental health problems can be expected to be higher in that group compared with nonmigrants.

The internal dropouts in the WHO (Ten) Mental Well-Being Index were replaced by imputation and the variable was analysed in several ways. Although there was no systemic dropout regarding any of the specific items, missing data were more prevalent for the WHO (Ten) Mental Well-Being Scale compared with questions using the Likert scale that was placed both immediately before and after the well-being index. This indicates that questions regarding mental health are sensitive and the missing data were not missing at random [43]. Through imputation, the sample size was increased. The proportions reporting high and low mental well-being before and after imputation did not change but a bias might have been introduced due to possible misclassification.

We do not have any information on dropout regarding work capacity but it can be expected that individuals with low mental work capacity refrain to a higher degree from participation in a questionnaire study with a large number of cognitively demanding questions [44]. If this is the case, the proportions reporting low work capacity might be low in comparison with the true distribution.

The choice of confounders in this study was mainly based on earlier research on CMD and sickness absence. It is well known that both vary with age, gender, education, socioeconomic position, marital status; and for sickness absence, also with hours worked and earlier sickness absence. An initial bivariate analysis of the associations between these variables and RTW was done and in most cases the non significant variables were excluded from further analyses. However, the basic covariate and gender were included even though the associations were non-significant in the bivariate analyses to keep these factors under control in different sub analyses.

## Conclusions

In this study, self-assessed persistent mental illness, low mental well-being and four dimensions of low work capacity increased the likelihood of late RTW in persons on all-cause sickness absence. This study is unique because it is based on new sick-leave spells and is the first to show that low mental well-being was an important determinant of late RTW even after adjustment for work capacity. Our findings support the importance of identifying individuals with low mental well-being as a way to prevent late RTW.

## Competing interests

The authors declare that they have no competing interests.

## Authors’ contributions

GH came up with the idea and the design of the overall project and is responsible for the content of the questionnaire. All authors participated in the design of this study. MB and JL performed the statistical analyses and drafted the manuscript. All authors contributed continuously to the manuscript throughout the writing process and read and approved the final manuscript.

## Pre-publication history

The pre-publication history for this paper can be accessed here:

http://www.biomedcentral.com/1471-244X/13/259/prepub
